# Efficacy and Safety of Manual Therapy in Haemophilic Ankle Arthropathy: A Randomised Crossover Clinical Trial

**DOI:** 10.3390/healthcare13172228

**Published:** 2025-09-05

**Authors:** Carlos Truque-Díaz, Raúl Pérez-Llanes, Javier Meroño-Gallut, Rubén Cuesta-Barriuso, Elena Donoso-Úbeda

**Affiliations:** 1 Faculty of Physiotherapy, Podiatry and Occupational Therapy, Catholic University San Antonio-UCAM, 30107 Murcia, Spain; ctruque@ucam.edu (C.T.-D.); edonoso@ucam.edu (E.D.-Ú.); 2 Department of Physiotherapy, Faculty of Medicine, University of Murcia, 30120 Murcia, Spain; rperez@um.es; 3 InHeFis Research Group, Instituto Asturiano de Investigación Sanitaria (ISPA), 33011 Oviedo, Spain; 4 Tú. Bienestar 360º, Physiotherapy and Medical Center, 30730 San Javier-Murcia, Spain; 5 Department of Surgery and Medical-Surgical Specialties, The Faculty of Medicine and Health Sciences, University of Oviedo, 33006 Oviedo, Spain

**Keywords:** haemophilia, physiotherapy, functionality, joint pain, range of motion, pressure pain threshold, kinesiophobia, postural stability

## Abstract

Background: Recurrent haemarthrosis leads to progressive and degenerative joint damage in patients with haemophilia from an early age. Haemophilic arthropathy is characterised by chronic pain, restricted range of motion, proprioceptive deficits, and structural alterations. The aim of this study was to evaluate the effectiveness of a manual therapy protocol in patients with haemophilic ankle arthropathy. Methods: A randomised, crossover, double-blind clinical trial was conducted. Thirteen patients with haemophilia were allocated to two sequences: A–B (intervention phase followed by placebo control) and B–A (placebo control followed by intervention). The intervention comprised joint mobilisation techniques, high-velocity low-amplitude manipulations, and myofascial release. In the placebo control condition, a simulated protocol was applied, consisting of intermittent contact and light pressure. Both conditions involved three physiotherapy sessions, delivered once weekly over three consecutive weeks. Outcome measures included functional capacity (2-Minute Walk Test), pain intensity (visual analogue scale), range of motion (goniometer), pressure pain threshold (algometer), joint status (Haemophilia Joint Health Score), kinesiophobia (Tampa Scale of Kinesiophobia), and postural stability (pressure platform). Following a four-week washout period, participants crossed over to the alternate condition. Results: No participants experienced ankle haemarthrosis or other adverse events during the intervention, confirming the safety of the protocol. Significant time*sequence interactions (*p* < 0.05) with high post hoc power (≥0.80) were observed for functional capacity, range of motion, and joint status. A significant sequence effect was also found for most clinical outcomes, with no evidence of a carry-over effect. Conclusions: This manual therapy protocol might be safe for patients with haemophilia. The physiotherapy intervention demonstrated improvements in functionality, range of motion, and joint status in individuals with haemophilic ankle arthropathy.

## 1. Introduction

Haemophilia is an X-linked congenital coagulopathy characterised by the deficiency or absence of one or more clotting factors. Depending on the factor affected, two forms are distinguished: haemophilia A (factor VIII deficiency) and haemophilia B (factor IX deficiency) [1]. The prevalence of haemophilia A has been estimated at 17 cases per 100,000 live births, and that of haemophilia B at 4 cases per 100,000 live births [2].

The main clinical manifestations of haemophilia are muscle and joint bleeding [3]. Recurrent haemarthrosis causes deposits of free iron in the joint, producing oxidative stress, which leads to chronic inflammation. Recurrent haemarthrosis leads to the accumulation of iron in the joint space, causing oxidative stress and chronic inflammation. This, in turn, promotes the release of cytokines that degrade collagen and cartilage. The inflammatory response may also induce angiogenesis and synovial hypertrophy, further elevating the risk of bleeding [4].

With repeated haemarthroses, a degenerative condition known as haemophilic arthropathy develops, most frequently affecting the ankles, knees, and elbows. This arthropathy is characterised by chronic pain, reduced mobility, muscle atrophy, and proprioceptive and biomechanical alterations. As a result, patients’ participation in daily activities and social life is diminished, with a marked impact on quality of life [5,6]. Prophylactic treatment, consisting of regular administration of deficient clotting factor concentrates or non-replacement therapies, remains the gold standard for preventing haemarthrosis.

Several studies have examined the safety of interventions such as myofascial therapy, passive mobilisation, and joint traction [7,8]. These techniques have also demonstrated benefits in reducing pain intensity and improving functionality, range of motion, and quality of life in adults with advanced arthropathy [9].

High-velocity, low-amplitude (HVLA) techniques, when combined with physical exercise, have been shown to improve mobility and reduce pain in patients with osteoarthritis [10]. In individuals with ankle instability, HVLA techniques have demonstrated efficacy in enhancing mobility, balance, and pain control [11]. However, only one pilot study has evaluated their safety in patients with haemophilia, reporting improvements in pain and ankle range of motion without adverse events [8].

Our previous work has investigated the efficacy of manual therapy in haemophilic ankle arthropathy, as well as differences in stability and balance parameters compared with healthy peers [6,8]. The present trial builds on this evidence by incorporating additional outcome measures, including joint status assessed using the Haemophilia Joint Health Score (HJHS), kinesiophobia, and pressure pain threshold, while specifically addressing the clinical safety of these interventions—an aspect that has been scarcely documented in this population. Furthermore, this study advances prior findings by employing a randomised, crossover, double-blind design with a larger sample size and by assessing both the safety and effectiveness of a comprehensive manual therapy protocol that includes HVLA techniques.

The aim of this study was to evaluate the efficacy of a manual therapy protocol comprising mobilisation, joint mobilisation techniques, HVLA techniques, and myofascial release in patients with haemophilic ankle arthropathy.

## 2. Materials and Methods

### 2.1. Design

A randomised, crossover, double-blind clinical trial with a follow-up period was conducted. The study adhered to the CONSORT 2025 statement for randomised controlled trials, including the specific recommendations for crossover designs [12].

### 2.2. Ethical Considerations

The study complied with the principles of the Declaration of Helsinki, and all participants provided written informed consent. Ethical approval was obtained from the Research Ethics Committee of Virgen de la Arrixaca University Hospital (ID: 2022-7-2-HCUVA). The trial was prospectively registered at ClinicalTrials.gov (identifier: NCT06816056; registration date: 10 February 2025).

### 2.3. Participants

The study was conducted between February and June 2025. Participants were recruited from the Malaga Haemophilia Association (Andalusia, Spain).

Inclusion criteria were as follows: (i) patients aged over 18 years; (ii) a medical diagnosis of haemophilia A; (iii) severe phenotype (<1% FVIII); (iv) a medical diagnosis of bilateral ankle arthropathy; and (v) a joint status score greater than 5 on the Haemophilia Joint Health Score (HJHS) [13]. Although the HJHS was used as the main inclusion criterion, we acknowledge its limited sensitivity for detecting longitudinal changes, as well as the heterogeneity in the clinical interpretation of its variations, as has been recently reported [14]. For this reason, the HJHS was complemented with more sensitive tools, including functional assessment (2MWT), range of motion, pain evaluation, and stabilometric measures, in order to achieve a more accurate characterisation of patients’ joint and functional status.

Patients were excluded if they (i) did not present ankle pain; (ii) had cognitive impairments that limited their ability to understand the assessments; (iii) had a diagnosis of epilepsy or severe visual disorders that precluded accurate assessment of postural stability; or (iv) were receiving physiotherapy treatment during the study period.

Patients with inhibitors were also included, as they represent a clinically relevant subgroup of individuals with severe haemophilia A (estimated prevalence: 20–30%). All participants with inhibitors were clinically stable under prophylaxis with bispecific monoclonal antibodies, had no contraindications to physiotherapy interventions, and were closely monitored throughout the study to ensure safety. All patients continued their previously prescribed pharmacological treatment regimen.

A total of 13 patients were enrolled in the study. The median age was 42 years (interquartile range: 14). In all participants, the ankle was identified as the target joint, as every patient presented with bilateral haemophilic ankle arthropathy confirmed by clinical diagnosis. Given this homogeneity, no differential responses to the manual therapy protocol were observed, since the intervention was consistently applied to the ankle joint in all cases.

### 2.4. Sample Size

The sample size was estimated using an a priori power analysis with G*Power software (v3.1.9.7). The statistical design corresponded to a mixed repeated-measures ANOVA with two sequences and six measurements, using the detection of a significant Time * Sequence interaction as the primary reference.

Previous trials have reported large effect sizes for functional outcomes in musculoskeletal disorders, such as a 5.52 m improvement in the 2-Minute Walk Test among patients with knee osteoarthritis [15]. However, to avoid overestimating the expected effect in a rare disease population, we adopted a more conservative effect size (f = 0.40). This strategy provided a more reliable power estimate and reduced the risk of type I error while maintaining adequate sensitivity to detect clinically meaningful changes in patients with haemophilic arthropathy, as previously observed in pilot physiotherapy studies [7,8]. Thus, 12 patients with haemophilia were required for the study.

### 2.5. Procedure

**Figure 1:**
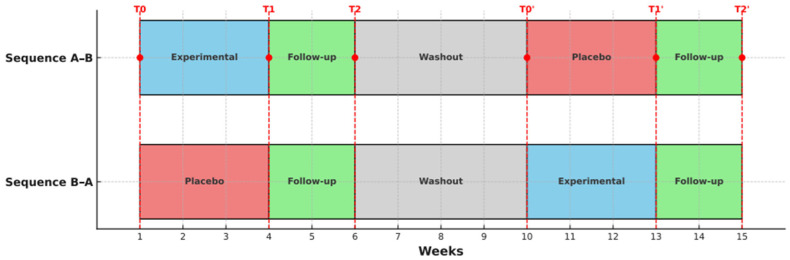
Crossover trial design.

The study consisted of two phases, with three assessments conducted in each phase: at baseline (T0), post-intervention (T1), and after a two-week follow-up (T2). Prior to the start, participants were randomly assigned to one of two sequences: A–B (experimental–placebo control) or B–A (placebo control–experimental). The first phase lasted seven weeks, followed by a four-week washout period to allow the effects of the previous treatment to dissipate, thereby minimising the risk of residual effects influencing subsequent results. In the second phase, the treatment condition (experimental vs. placebo control) was reversed for both sequences. To aid understanding of the study design, Figure 1 illustrates the structure of the randomised crossover trial, including the sequence of interventions, the washout period, and the timing of assessments.

### 2.6. Outcome Measures

The primary outcome was functional capacity. Secondary outcomes included pain intensity, range of motion, pressure pain threshold, joint status, kinesiophobia, and postural stability.

Functional capacity was assessed using the 2-Minute Walk Test, a validated and modified version of the 6-Minute Walk Test. The test was carried out in a 30-m-long indoor corridor marked out with cones. Patients walked back and forth along the corridor, and the total distance covered in metres was recorded. Patients were instructed to walk as fast as possible without running. The assessor recorded the distance covered in m. This test has demonstrated high reliability (ICC = 0.82) [16].

Pain intensity was assessed using a visual analogue scale (VAS). On a 10 cm line, patients indicated the intensity of ankle pain, where 0 represented “no pain” and 10 the “worst pain imaginable”. The VAS has demonstrated excellent reliability (ICC = 0.99) in patients with chronic musculoskeletal pain [17].

The range of motion of the ankle was measured. An analogue goniometer was used with its fixed arm aligned to the fibula and the movable arm aligned to the fifth metatarsal. Evaluators ensured standardised positioning to minimise compensatory movements [18]. Range of motion was assessed with a goniometer, expressed in degrees. This instrument has demonstrated high reliability (ICC = 0.85–0.96) for the evaluation of ankle mobility [19].

The pressure pain threshold was measured using a pressure algometer (model Wagner FDIX, Wagner Instruments, Greenwich, CT, USA). The evaluator applied continuous pressure to the ventral aspect of the medial and lateral malleoli until the patient first perceived pain [20]. This measure of pain threshold has demonstrated excellent reliability (ICC = 0.99) [21].

Joint status was assessed using the Haemophilia Joint Health Score (HJHS) [13]. This additive scale, specific to patients with haemophilia, evaluates joint condition in the knees, ankles, and elbows. It consists of eight items: swelling and duration of swelling, pain, muscle atrophy and strength, crepitus, and loss of flexion and extension. The HJHS has shown high reliability (α = 0.88) [13]. The scoring range per joint is 0–20 points.

Kinesiophobia was measured using the Tampa Scale of Kinesiophobia [22]. This questionnaire assesses fear of movement in individuals with musculoskeletal disorders and comprises 11 items. It has demonstrated high reliability (ICC = 0.77–0.99) [23]. The total score ranges from 11 to 44 points.

Postural stability was assessed using a pressure platform (model RsScan^®^, Paal, Belgium) in combination with the FootScan^®^ (Paal, Belgium) pressure measurement system. This device measures plantar pressure through an X–Y matrix of sensitive resistive sensors and records data while the participant is standing or walking on the platform. Assessments were conducted using the basic 0.5 m platform equipped with 4096 resistive sensors and a data acquisition frequency of 300 Hz. This system has demonstrated good reliability (ICC = 0.81–0.86) [24].

Postural stability and balance were analysed with eyes open and closed for 30 s. Displacements along the X and Y axes were measured in mm while the area of the stabilometric ellipse was measured in mm^2^.

Prior to the start of the trial, a pilot analysis was conducted to assess intra-observer reliability. The variables were evaluated in six individuals who were not included in the main study. Excellent reliability was observed for functional capacity (ICC = 0.99; SEM = 1.70) and maximum posterior displacement (ICC = 0.99; SEM = 0.05). High reliability was found for range of motion (ICC = 0.93; SEM = 3.12), external malleolar pain threshold (ICC = 0.92; SEM = 7.73), stability area (ICC = 0.87; SEM = 0.20), and maximum anterior displacement (ICC = 0.87; SEM = 0.09). Moderate reliability was observed for the internal malleolar pain threshold (ICC = 0.80; SEM = 10.25).

### 2.7. Intervention

In both conditions, a weekly 50 min session was delivered over three consecutive weeks. The experimental manual therapy protocol was specifically designed for patients with haemophilic ankle arthropathy. Techniques were applied in a standardised and homogeneous manner across all participants to minimise intervention variability and ensure reproducibility. Although in clinical practice the choice of techniques is usually tailored to the individual restrictions of each patient, in this study a uniform protocol was applied, as all participants presented with dorsiflexion limitation and ankle mobility restriction. This approach allowed the observed effects to be more confidently attributed to the intervention.

The same number of techniques were performed in both sequences, over the same period, and with the same preparatory manoeuvre. However, participants assigned to the placebo control condition did not receive any passive joint mobilisation through wide ranges of motion, joint manipulations, or myofascial induction techniques. Instead, the manoeuvres consisted of intermittent pressure contact but were delivered with identical timing, repetitions, and positioning to those of the experimental intervention. For example, during talocrural mobilisation into dorsiflexion, the patient was positioned supine with the knee extended, while the therapist applied a posteroanterior glide to the talus using rhythmic oscillations for 30 s. Similarly, in the myofascial technique applied to the triceps surae, sustained and progressive pressure was delivered for 90 s at the proximal region of the Achilles tendon. Table 1 summarises the main characteristics of the interventions performed in both conditions.

The interventions were administered by two physiotherapists specialising in manual therapy, both with experience in the management of haemophilic ankle arthropathy. Participants were blinded to their assigned sequence and remained unaware of which intervention they were receiving at any point during the study.

### 2.8. Randomisation and Blinding

Patients were randomised into the two treatment sequences using a permuted block design. The individual responsible for randomisation was blinded to participant identity, study objectives, and treatment allocation.

Participants were also blinded throughout the study and did not know which intervention corresponded to the experimental condition or the placebo control. All assessments were conducted by the same evaluator, who was blinded to both the intervention condition and the treatment sequence. The evaluator only had access to the numerical code assigned to each participant, and evaluations were performed in no pre-established order.

### 2.9. Statistical Analysis

Statistical analyses were performed using SPSS software for Windows, version 26.0 (IBM Corp., Armonk, NY, USA). Intra-observer reliability was assessed using the two-way random intraclass correlation coefficient.

The sequence, period, and carryover effects of the crossover design were evaluated using *t*-tests, given the small sample size and data structure. The sequence effect was analysed using an independent *t*-test on the pre–post change in Phase 1, comparing conditions according to sequence (A–B vs. B–A). The period effect was assessed with a paired *t*-test comparing changes between Phases 1 and 2 within each participant. The carryover effect was estimated by comparing baseline values at the start of Phase 2 between sequences using an independent samples *t*-test. This approach has been validated and recommended for small-sample studies, where mixed-model methods may be overfitted or lack sufficient statistical power [25].

The primary analyses focused on within-subject comparisons between the experimental and placebo control conditions, with each participant serving as their own control. Although linear mixed-effects models are generally recommended for crossover trials with repeated measures, in this study a repeated-measures ANOVA was used for methodological reasons. First, the small sample size limited the reliability of mixed models, which require larger datasets to estimate random effects and covariance structures with accuracy. Second, the dataset was nearly complete and balanced across conditions and time points, minimising one of the main advantages of mixed models (their robustness in handling missing data). Moreover, the experimental design, with clearly differentiated conditions (experimental vs. placebo control) and a counterbalanced sequence (A–B vs. B–A), was well suited to the factorial framework of repeated-measures ANOVA, allowing for clear analysis of intra- and inter-subject effects. When the assumption of sphericity was violated, Greenhouse–Geisser corrections were applied.

An intention-to-treat analysis was performed using the carryover observation method. Statistical significance was set at *p* < 0.05 with a 95% confidence interval.

## 3. Results

**Figure 2:**
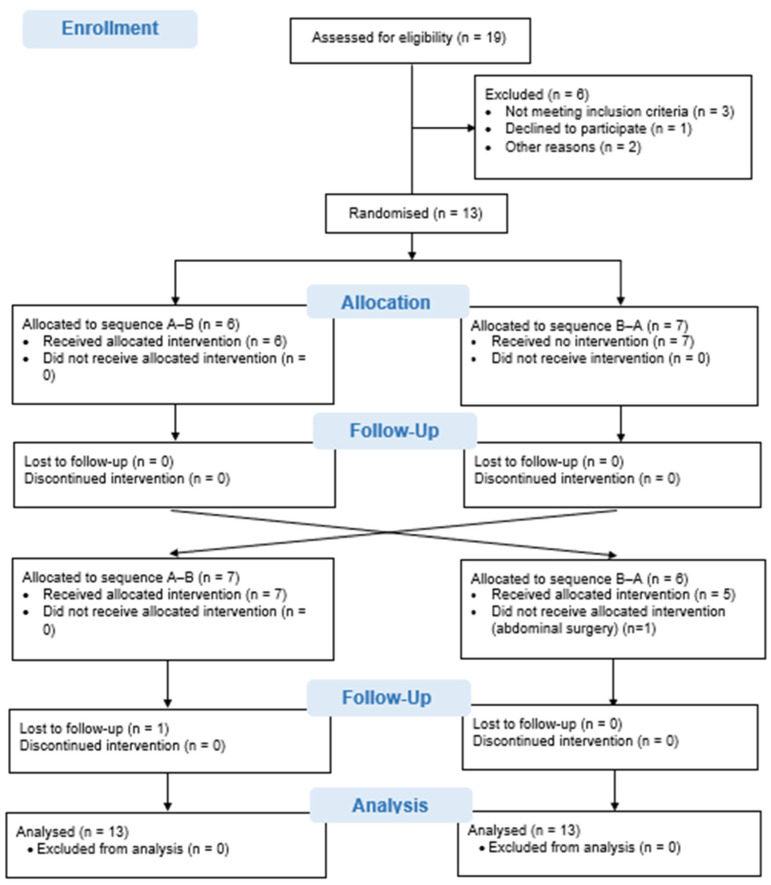
CONSORT flow chart.

Thirteen patients with haemophilia and bilateral ankle arthropathy were included in the study. During the trial, two participants withdrew: one in the second phase due to abdominal surgery, and another who was unable to attend the second follow-up evaluation because of scheduling conflicts. Figure 2 presents the study flow chart.

### 3.1. Descriptive Analysis

The median age of participants was 42 years (interquartile range [IQR]: 14). All had a diagnosis of haemophilia A with a severe phenotype (<1% FVIII). The majority received prophylactic treatment (84.6%). Three participants (23.1%) had developed inhibitors and were receiving prophylaxis with bispecific monoclonal antibodies. Table 2 summarises the main characteristics of the study population.

Measures of central tendency (median) and dispersion (IQR) for all study variables, across sequences and time points, are provided in Supplementary Table S1. These detailed descriptive data are included as Supplementary Materials to enhance the transparency of the study and to facilitate their potential use in future systematic reviews and meta-analyses.

### 3.2. Safety of the Intervention

None of the participants developed clinical haemarthrosis, haematomas, or other adverse effects during the study as a result of the interventions received.

### 3.3. Analysis of Study Effects

In crossover clinical trials, it is essential to evaluate potential sequence, period, and carryover effects to ensure that observed differences are attributable to the intervention itself rather than to treatment order, timing of assessment, or residual effects from the preceding phase. The sequence effect assesses whether the order of interventions influences outcomes, the period effect captures changes related to time rather than treatment, and the carryover effect identifies whether residual effects from the first phase persist into the second. These analyses confirm that differences can be attributed to the intervention itself.

When comparing clinical change in Phase 1 between the two sequences (sequence effect), statistically significant differences (*p* < 0.05) were found for all variables except pain intensity (*p* = 0.13), kinesiophobia (*p* = 0.76), and stabilometric measurements with eyes open and closed (*p* > 0.05). Analysis of the period effect revealed no statistically significant differences (*p* > 0.05) between the changes observed in Phases 1 and 2 for any study variable, except for minimum anterior displacement (*y*-axis) and posterior displacement (*x*-axis) with eyes open and closed (*p* < 0.05), maximum posterior displacement with eyes open (*p* = 0.004), and maximum posterior displacement with eyes closed (*p* < 0.000). Analysis of the carryover effect showed no statistically significant differences (*p* > 0.05) in any study variable. Table 3 presents the results of the sequence, period, and carryover effect analyses.

The apparent differences observed in Phase 1 (sequence effect) can be attributed to the impact of the experimental physiotherapy intervention compared with the placebo control condition. In the analysis of the period effect, differences were detected only in certain stabilometric variables, which may reflect the time elapsed between the two phases rather than the intervention itself, suggesting a process of patient adaptation. Similarly, no carryover effects were identified in any of the evaluated variables, indicating that the effects of the previous intervention did not persist and that the washout period was adequate. Therefore, a full crossover repeated-measures analysis was performed.

### 3.4. Analysis of Repeated Measures

A statistically significant effect was observed across the six measurement points for functional capacity (F_[2,22]_ = 8.63; *p* = 0.002; ŋ^2^_p_ = 0.44) and joint status (F_[1.45,34.88]_ = 5.53; *p* = 0.01; ŋ^2^_p_ = 0.18). In the stabilometric analysis, significant effects were found for minimum posterior displacement with eyes open (F_[2,22]_ = 10.78; *p* = 0.001; ŋ^2^_p_ = 0.49) and closed (F_[2,22]_ = 15.35; *p* < 0.001; ŋ^2^_p_ = 0.58), maximum anterior displacement with eyes open (F_[2,22]_ = 4.61; *p* = 0.02; ŋ^2^_p_ = 0.29) and closed (F_[2,22]_ = 9.27; *p* = 0.001; ŋ^2^_p_ = 0.45), and minimum anterior displacement with eyes open (F_[2,22]_ = 4.87; *p* = 0.01; ŋ^2^_p_ = 0.30).

When examining differences between the experimental and placebo control sequences, statistically significant effects were observed only for maximum posterior displacement with eyes open (F = 4.87; *p* = 0.049; ŋ^2^_p_ = 0.31).

For the Time * Sequence interaction, significant differences were detected in functional capacity (F_[2,22]_ = 6.06; *p* = 0.01; ŋ^2^_p_ = 0.35), joint status (F_[2,48]_ = 3.93; *p* = 0.02; ŋ^2^_p_ = 0.14), and range of motion (F_[1.20,28.94]_ = 5.67; *p* = 0.02; ŋ^2^_p_ = 0.19).

**Figure 3:**
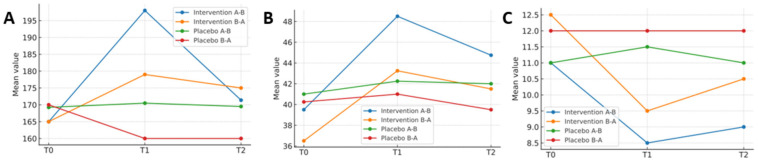
Line graphs showing mean values at baseline (T0), post-intervention (T1), and follow-up (T2) for each treatment sequence (A–B and B–A) in both the intervention and placebo phases. (**A**) Functional capacity (metres). (**B**) Range of motion (degrees). (**C**) Joint status (HJHS, 0–20 scale).

Table 4 presents the results of the repeated-measures analysis. To complement the tabular presentation, graphical representations were included to illustrate the evolution of the main variables over time. Figure 3 shows changes in functional capacity, range of motion, and joint status across the intervention and placebo phases for both treatment sequences.

## 4. Discussion

This study aimed to evaluate the efficacy of a manual therapy protocol in patients with haemophilic ankle arthropathy. The protocol demonstrated beneficial effects on functional capacity, range of motion, and joint status when compared with placebo treatment. In contrast, both interventions produced similar changes in joint pain intensity, pressure pain thresholds, and kinesiophobia.

Beyond statistical significance, it is essential to consider the clinical relevance of the observed changes. Recent evidence suggests that an improvement of ≥4 points in the total Haemophilia Joint Health Score (HJHS) and ≥2 points in an individual joint can be regarded as clinically meaningful [14]. In our cohort, the improvements approached these thresholds, supporting the interpretation that the intervention may exert clinically relevant effects on ankle joint health. Moreover, in the context of rare diseases such as haemophilia, even modest changes in functional capacity or joint mobility may translate into perceptible improvements in quality of life and participation in daily activities.

In our study, functional capacity improved in patients who received manual therapy compared with placebo, increasing their ability to walk longer distances within the same time frame. Loss of joint range of motion, together with pain, a characteristic feature of arthropathy, reduces lower-limb functional capacity [6], thereby affecting ambulation and gait [26]. A direct correlation has been reported between the maximum angle of ankle dorsiflexion and lower-limb biomechanics during walking [27]. Alterations in gait patterns may be associated with impaired physical performance, and walking performance is closely linked to lower-limb muscle strength. The relationship between range of motion, muscle strength, and gait suggests that improving ankle mobility may facilitate more efficient walking. Clinically, this translates into an enhanced ability to perform everyday activities such as walking or climbing stairs [28]. Furthermore, ankle mobility correlates with subjective perceptions of functionality, such that improvements in ankle mobility positively influence patients’ perceptions of their capacity to move and function in daily life [29].

Several studies have evaluated improvements in range of motion following manual therapy techniques in patients with haemophilic arthropathy [8,30]. To our knowledge, this is the first study to incorporate joint manipulation and high-velocity, low-amplitude (HVLA) techniques in patients with haemophilia, where we observed significant changes in those receiving the intervention compared with placebo. Manual stimulation of tissues may enhance tolerance to stress and induce hypoalgesia in the treated area. Mechanistically, manual therapy is thought to activate descending inhibitory pathways and modulate afferent input, resulting in hypoalgesia and increased tissue compliance [31], which may explain the improvements in range of motion observed in this trial. Nevertheless, it should be noted that in the lower extremity, increases in range of motion of less than five degrees may not be considered clinically meaningful, as reported in standard methodological references [19]. For this reason, interpretation of our findings requires careful consideration of both statistical significance and clinical magnitude. The changes observed in joint status are comparable to those reported with other manual therapy techniques [30]. These improvements may be attributed to a combination of direct effects on periarticular tissues. Notably, normalisation of tension and improvements in joint biomechanics—through increased range of motion, reduced joint stress, enhanced vascularisation, and modulation of biological processes such as inflammation—may have contributed. The normalisation of tension in the periarticular connective system, achieved via improvements in ankle range of motion, may enhance biomechanics during functional activities. This, in turn, could reduce mechanical joint stress, help prevent further deterioration, and promote better joint health [32]. Beyond statistical significance, the clinical relevance of changes in the HJHS should also be considered. Recent evidence indicates that improvements of ≥4 points in the total score and ≥2 points in an individual joint may represent acceptable thresholds of clinical benefit [14]. In this context, the improvements observed in our cohort approached these thresholds, supporting the interpretation that the intervention may exert clinically relevant effects on ankle joint health. Manual therapy may optimise joint health through mechanisms such as modulation of the autonomic nervous system and increased plasma levels of nitric oxide. These effects enhance vascularisation and nutrition of joint tissues. Consequently, they may promote increased physical activity, which is in turn associated with the regulation of inflammatory biomarkers, stimulation of bone turnover, and enhancement of the antioxidant response. Collectively, these biological changes may support the integrity and functionality of joint tissues, contributing to improved joint health and the prevention of degenerative disease [33].

The combination of analgesic therapies with specific stimuli can induce pain-inhibitory responses [34]. Neuroimaging studies have identified cortical and cerebellar circuits involved in pain reduction under placebo conditions [35]. Pain experienced by patients with haemophilic arthropathy can be considered mixed nociceptive/neuropathic. In this type of pain, local nociceptive mechanisms linked to histological damage and inflammation coexist with peripheral and central sensitisation phenomena associated with persistent pain [36]. The multifactorial nature of persistent pain in these patients, all of whom received a manual stimulus (placebo or therapeutic), may explain the observed changes in pain modulation. These changes could be mediated by local, spinal, and supraspinal neurobiological mechanisms and influenced by contextual factors such as expectations, beliefs, and values. Such mechanisms may contribute to the improvements perceived in pain scores [37]. Finally, positive expectations—shaped by information and belief in the treatment—are central drivers of the placebo effect. These develop through associative learning, in which neutral stimuli are linked to prior experiences of pain relief, thereby conditioning analgesic responses. In addition to conditioning, placebo responses may also be reinforced by patient expectations and contextual cues, such as therapist–patient interactions and the clinical environment, which can modulate symptom perception and treatment outcomes. Previous positive experiences with effective interventions may, in turn, amplify the placebo effects of subsequent treatments [38].

Regardless of whether participants received manual therapy or a placebo, changes were observed in kinesiophobia. As with pain intensity, the stimulus generated by the intervention—whether active or sham—may have elicited a positive response in relation to fear of movement. Catastrophic thoughts about pain have been associated with kinesiophobia [39]. Thus, the improvements in pain perception observed in both groups may explain the reductions in fear of movement. Patients with advanced arthropathy typically present with slightly impaired postural stability compared with healthy counterparts [6]. In individuals with haemophilia, variations in the ellipse area are smaller, indicating excessive postural stability. In our study, no differences were found between the two interventions in this regard. In patients with haemophilic arthropathy, abnormal responses may arise from negative beliefs, catastrophic thinking, and, above all, kinesiophobia. This fear of movement can lead to altered motor patterns, such as slower movements, avoidance of specific tasks, or the adoption of rigid postures [40].

It should be noted that the duration of the intervention—three weekly sessions—was relatively short, which may limit the generalisability of the findings. However, the study was deliberately designed with a cautious timeframe to ensure patient safety, particularly given the novelty of including high-velocity, low-amplitude (HVLA) techniques in patients with haemophilia. Previous studies employing fascial therapy and joint mobilisation in haemophilic arthropathy have likewise reported positive effects after short intervention periods [7,8,30]. Similarly, in other musculoskeletal conditions such as knee osteoarthritis and chronic ankle instability, short-term manual therapy protocols have been associated with meaningful improvements in pain and mobility [10,11]. Our results are therefore consistent with the existing literature, suggesting that even brief interventions may elicit clinically relevant benefits, although longer-term trials are needed to confirm the persistence and magnitude of these effects.

Our findings are consistent with those of Donoso-Úbeda et al. [30], who observed significant improvements in functional capacity and joint status following a structured physiotherapy programme in patients with haemophilic arthropathy. Likewise, Truque-Díaz et al. [8] demonstrated that manual therapy interventions reduced pain and improved mobility in haemophilic patients without reporting adverse events. Taken together, these results and those of the present trial support the role of physiotherapy—particularly manual therapy protocols—as a safe and beneficial strategy to enhance function and joint health in haemophilia. Importantly, the specific inclusion of HVLA techniques in our study provides novel evidence that complements and extends previous findings. Although soft tissue release, joint mobilisation, and manipulation differ in their mechanisms of action—and some may be contraindicated at certain stages of haemophilia—in this trial they were applied exclusively in patients with chronic ankle arthropathy who were clinically stable and free from acute haemarthrosis at the time of intervention. It should also be noted that not all participants were receiving conventional prophylaxis; however, those with inhibitors were treated with a bispecific antibody, which enabled the protocol to be applied safely. The aim of the trial was not to assess each technique in isolation, but rather to evaluate the overall efficacy and safety of a combined manual therapy protocol reflecting routine clinical practice. Future studies should seek to disentangle the specific contributions and differential effects of individual techniques.

Post hoc analysis of statistical power (1 – β) can be particularly valuable in studies with small samples and rare clinical conditions such as haemophilia. In our trial, post hoc power values exceeding 0.80 were observed for functional capacity, range of motion, and joint status, reinforcing the robustness of these findings. In conclusion, this randomised crossover clinical trial represents a relevant methodological advance by applying a longitudinal analytical framework and an expanded set of outcome measures—including clinical (HJHS), functional (2MWT, ROM), psychometric (kinesiophobia), and physiological parameters (pressure pain threshold, stabilometry)—alongside a systematic safety evaluation. Together, these elements provide a more comprehensive and multidimensional characterisation of the response to manual therapy in haemophilic ankle arthropathy. Nonetheless, the results should be interpreted with caution and contextualised within the specific design and objectives of the study [41].

### Limitations of the Study

This study has several limitations that should be acknowledged. The principal limitation is the small sample size. Although the number of participants was based on an a priori power calculation and achieved the required statistical power, the prevalence of severe haemophilia A with ankle arthropathy is low, making recruitment particularly challenging. Nevertheless, the methodological strengths of the study—namely the randomised, double-blind, crossover design—helped reduce bias and enhance the reliability of the findings despite the restricted sample size. The single-centre recruitment may also limit external validity, as the results may not fully capture the heterogeneity of patients across different clinical settings. Furthermore, the short intervention period of only three sessions restricts the ability to determine whether longer treatment durations could yield greater or more sustained benefits. Although only patients with severe haemophilia A were included, the results should be generalised with caution to individuals with other haemophilia phenotypes or to those not receiving prophylaxis.

Another relevant limitation concerns the heterogeneity in joint status (acute vs. chronic stages), as treatment response may vary according to disease stage. Although chronic ankle arthropathy predominated in our sample, this variability could not be completely avoided. Future research should consider stratification by joint stage and the use of complementary imaging and clinical assessment methods.

## 5. Conclusions

A physiotherapy protocol comprising mobilisation, joint mobilisation techniques, high-velocity low-amplitude techniques, and myofascial release is safe for patients with haemophilia. Manual therapy can improve functionality, range of motion, and joint status in individuals with ankle arthropathy. For perceptual variables such as pain and fear of movement, both therapeutic manual interventions and placebo stimuli produced similar changes. Therefore, the results of this trial should be interpreted from both statistical and clinical perspectives, recognising that not all statistically significant differences necessarily translate into clinically meaningful outcomes.

## Figures and Tables

**Table 1:** Description of the intervention carried out in both conditions.

Manoeuvre	Group	Position	Description	Duration (min)
Preparatory	Experimental	Supine position	Active plantar and dorsal ankle flexion exercise.	2
	Control		Very short-range plantar movement.	
Global passive mobilisation	Experimental	Supine position	Twenty passive mobilisations of the forefoot and midfoot.	2
	Control		Twenty intermittent light pressure contacts on the medial and lateral malleolus.	
Calcaneocuboid mobilisation	Experimental	Supine position	Twenty mobilisations of the calcaneocuboid joint; functional and structural work.	3
	Control		Twenty intermittent light pressure contacts on the calcaneus.	
Talonavicular mobilisation	Experimental	Supine position	Twenty mobilisations of the talonavicular joint; functional and structural work.	3
	Control		Twenty intermittent light pressure contacts on the talonavicular area.	
Talus manipulation	Experimental	Supine position	Posterior talus manipulation.	1
	Control		Continuous light contact on the talonavicular area.	
Tibial manipulation displacement	Experimental	Supine position	Ten anterior-posterior tibial displacement mobilisations.	2
	Control		Intermittent light pressure contacts on the distal anterior tibial area.	
Tibiotalar decompression	Experimental	Supine position	Two high-velocity, short-stroke, very gentle tibiotalar manipulations.	1
	Control		Continuous light contact on the talonavicular area.	
Plantar fascia induction	Experimental	Supine position	Fascial induction technique for plantar fascia foot receptors.	3–5
	Control		Intermittent light pressure contacts on the plantar area.	
Sustained traction (unwinding)	Experimental	Supine position	Sustained tibiotalar traction, stimulation of capsuloligamentous components.	3–5
	Control		Intermittent light pressure contacts on the medial and lateral malleolus.	
Triceps surae induction	Experimental	Prone position	Fascial induction on the triceps surae.	3–5
	Control		Intermittent light pressure contacts on the triceps surae.	

**Table 2:** Descriptive analysis, median, and interquartile range of patients included in the study in the pre-phase 1 assessment.

Variables			All Samples (*n* = 13)	Sequence B–A (*n* = 7)	Sequence A–B (*n* = 6)
Age (years)			42 (14)	39 (16)	43.5 (22.25)
Weight (kg)			85 (25.5)	87 (26)	75 (29.25)
Height (cm)			173 (15.5)	173 (31)	177 (13.75)
			*n* (%)	*n* (%)	*n* (%)
Treatment	Prophylaxis		11 (84.6)	6 (85.7)	5 (83.3)
	On demand		2 (15.4)	1 (14.3)	1 (16.7)
Development of inhibitors		Yes	3 (23.1)	2 (28.6)	1 (16.7)
		No	10 (76.9)	5 (71.4)	5 (83.3)
Need for orthoses in activities of daily living		Yes	1 (7.7)	1 (14.3)	0 (0)
		No	12 (92.3)	6 (85.7)	6 (100)

Sequence B–A: sequence placebo control–intervention; Sequence A–B: sequence intervention–placebo control; *n*: number of subjects; %: percentage.

**Table 3:** Results of the sequence, period, and carryover effect analyses.

Variables	Sequence Effect	Period Effect		Carryover Effect	
	MD [95% CI]	t	M [95% CI]	t	MD [95% CI]
Functional capacity	28.44 [20.26; 36.62] **	0.35	2.26 [−11.80; 16.33]	0.02	0.28 [−38.77; 39.34]
Range of motion	8.78 [6.39; 11.17] **	0.11	0.16 [−2.90; 3.22]	1.10	5.25 [−4.55; 15.05]
Joint damage	−2.39 [−2.84; −1.94] **	−0.14	−0.07 [−1.17; 1.02]	−1.21	−0.96 [−2.59; 0.66]
Joint pain	−0.85 [−2.01; 0.29]	−0.26	−0.08 [−0.78; 0.61]	−0.29	−0.19 [−1.52; 1.14]
Pressure pain threshold (internal malleolus)	12.48 [1.89; 23.06] *	0.08	0.25 [−6.35; 6.86]	1.44	11.19 [−4.82; 27.20]
Pressure pain threshold (external malleolus)	12.96 [2.63; 23.30] *	0.67	1.97 [−4.06; 8.01]	0.45	3.03 [−10.89; 16.97]
Kinesiophobia	0.95 [−5.90; 7.80]	0.26	0.69 [−5.03; 6.42]	−0.27	−1.16 [−10.38; 9.04]
Min-X with open eyes	2.0 [−4.60; 8.60]	−3.61	−5.31 [−8.51; −2.10] *	0.004	0.01 [−5.97; 5.99]
Min-Y with open eyes	3.01 [−2.12; 8.15]	2.61	3.92 [0.65; 7.18] *	−0.12	−0.19 [−3.44; 3.06]
Max-X with open eyes	−2.22 [−7.38; 2.93]	−3.50	−10.03 [−16.27; −3.79] *	0.96	6.54 [−8.42; 21.52]
Max-Y with open eyes	0.15 [−4.92; 5.23]	0.43	1.31 [−5.29; 7.91]	0.73	4.03 [−7.99; 16.06]
Area with open eyes	−6.51 [−15.26; 2.24]	−1.35	−16.81 [−43.82; 10.21]	1.07	30.82 [−27.40; 89.04]
Min-X with closed eyes	−0.55 [−5.06; 3.94]	−4.19	−5.38 [−8.17; −2.59] *	−0.18	−0.55 [−7.07; 5.95]
Min-Y with closed eyes	−1.11 [−4.78; 2.55]	3.88	4.96 [2.18; 7.74] *	−0.22	−0.45 [−4.87; 3.96]
Max-X with closed eyes	2.25 [−11.33; 15.83]	−2.16	−6.19 [−12.42; 0.04]	0.37	1.02 [−4.93; 6.97]
Max-Y with closed eyes	−0.45 [−4.88; 3.98]	4.72	6.53 [3.52; 9.55] **	0.54	1.13 [−3.45; 5.71]
Area with closed eyes	3.83 [−13.99; 21.56]	−1.23	−3.69 [−10.23; 2.84]	1.11	9.02 [−8.78; 26.83]

t: t-student statistic; MD: means difference; M: mean; 95% CI: 95% confidence interval. ** Significant difference between improvements of the study groups (*p* < 0.01). * Significant difference between improvements of the study groups (*p* < 0.05).

**Table 4:** Repeated-measures analysis statistics.

Variables		Time Effect		Sequence Effect		Time * Sequence Interaction		
		F	ŋ^2^_p_	F	ŋ^2^_p_	F	ŋ^2^_p_	Power (1–β)
Joint structure	Functional capacity	8.63 *	0.44	1.41	0.11	6.06 *	0.35	0.99
	Range of motion	3.07	0.11	1.10	0.04	5.67 *	0.19	0.95
	Joint damage	5.53 *	0.18	0.40	0.02	3.93 *	0.14	0.85
Pain	Joint pain	2.58	0.09	0.52	0.02	0.23	0.01	0.10
	Pressure pain threshold (internal malleolus)	0.08	0.03	0.02	0.01	0.67	0.03	0.23
	Pressure pain threshold (external malleolus)	2.32	0.09	3.02	0.11	2.21	0.08	0.57
Kinesiophobia		1.47	0.11	2.01	0.15	1.18	0.09	0.63
Stabilometric analysis	Min-X with open eyes	10.78 *	0.49	0.70	0.06	1.61	0.12	0.78
	Min-Y with open eyes	4.87 *	0.30	1.74	0.13	0.45	0.04	0.30
	Max-X with open eyes	2.01	0.15	4.87 *	0.31	0.43	0.04	0.30
	Max-Y with open eyes	4.61 *	0.29	0.002	0.00	0.15	0.01	0.10
	Area with open eyes	1.97	0.15	1.29	0.10	1.43	0.11	0.73
	Min-X with closed eyes	15.35 **	0.58	0.00	0.00	0.03	0.003	0.06
	Min-Y with closed eyes	1.79	0.14	4.40	0.28	0.66	0.05	0.37
	Max-X with closed eyes	0.82	0.07	1.00	0.08	0.51	0.04	0.30
	Max-Y with closed eyes	9.27 *	0.45	0.02	0.002	0.32	0.03	0.23
	Area with closed eyes	2.47	0.18	1.79	0.14	0.04	0.004	0.07

F: Fisher–Snedecor statistic; ES: effect size (ŋ^2^_p_: partial square eta); power (1–β): post hoc power calculated with effect size (f). ** Significant difference between improvements of the study groups (*p* < 0.01). * Significant difference between improvements of the study groups (*p* < 0.05).

## Data Availability

The data that support the findings of this study are available on request from the corresponding author.

## Supplementary Materials

The following supporting information can be downloaded at: https://www.mdpi.com/article/10.3390/healthcare13172228/s1, Table S1: Central tendency (median) and dispersion (interquartile range) statistics for the study variables, in both sequences, in the different assessments.
