# Supplementary material for: Efficacy and Safety of Manual Therapy in Haemophilic Ankle Arthropathy: A Randomised Crossover Clinical Trial
Source: Healthcare (Basel). 2025 Sep 5;13(17):2228. doi: 10.3390/healthcare13172228 (PMC12428218; doi:10.3390/healthcare13172228)
Supplement: Supplementary file 1 [file healthcare-13-02228-s001.zip › healthcare-3750084-supplementary.pdf]

Supplementary Table S1. Central tendency (median) and dispersion (interquartile range) statistics for the study variables, in both sequences, in the different assessments.

.

Variables	Condition (sequences)	T0	T1	T2
Functional capacity (m)	Intervention phase (sequence A-B)	165 (59.75)	198 (60.5)	171.38 (56.25)
	Intervention phase (sequence B-A)	165 (40)	179 (41.5)	175 (52)
	Placebo control phase (sequence A-B)	169.25 (61.25)	170.5 (61.38)	169.5 (59.3)
	Placebo control phase (sequence B-A)	170 (60)	160 (51.2)	160 (55)
Range of motion (degrees)	Intervention phase (sequence A-B)	39.5 (19.13)	48.5 (20.38)	44.75 (19.38)
	Intervention phase (sequence B-A)	36.5 (14.25)	43.25 (17.63)	41.5 (15)
	Placebo control phase (sequence A-B)	41 (22.5)	42.25 (23)	42 (23)
	Placebo control phase (sequence B-A)	40.25 (19.25)	41 (18.88)	39.5 (20.75)
Joint damage (0 - 20)	Intervention phase (sequence A-B)	11 (3)	8.5 (3)	9 (3.75)
	Intervention phase (sequence B-A)	12.5 (4)	9.5 (4)	10.5 (4.25)
	Placebo control phase (sequence A-B)	11 (3)	11.5 (3)	11 (2.75)
	Placebo control phase (sequence B-A)	12 (3.25)	12 (4)	12 (3.25)
Joint pain (0 - 10)	Intervention phase (sequence A-B)	3 (2.23)	1.15 (1.95)	1 (1.45)
	Intervention phase (sequence B-A)	3.65 (2.15)	2.1 (1.93)	2.05 (1.68)
	Placebo control phase (sequence A-B)	3.85 (1.88)	3.4 (1.7)	3.05 (1.73)
	Placebo control phase (sequence B-A)	3.25 (2.8)	2.85 (1.73)	2.8 (2.25)
Internal malleolus pressure pain threshold (N)	Intervention phase (sequence A-B)	63.87 (33.06)	66.52 (33.65)	69.07 (34.93)
	Intervention phase (sequence B-A)	47.97 (20.81)	57.77 (20.8)	54.4 (23.73)
	Placebo control phase (sequence A-B)	63.8 (38.13)	67.07 (35.43)	67.87 (34.94)
	Placebo control phase (sequence B-A)	45.3 (32.48)	45.5 (22.16)	45.4 (33.51)
External malleolus pressure pain threshold (N)	Intervention phase (sequence A-B)	58.3 (18.05)	68.05 (25.4)	66.35 (20.49)
	Intervention phase (sequence B-A)	50.42 (18.46)	59.65 (19.23)	56.5 (13.43)
	Placebo control phase (sequence A-B)	55.85 (28)	57.37 (19.71)	59.47 (24.94)
	Placebo control phase (sequence B-A)	41.3 (17.83)	43.6 (23.21)	43.67 (29.58)
Kinesiofobia	Intervention phase (sequence A-B)	25 (8)	23.5 (4.75)	23 (8.5)
	Intervention phase (sequence B-A)	29 (8)	27 (7)	28 (5)
	Placebo control phase (sequence A-B)	24.5 (18)	21 (10.25)	23 (10.5)
	Placebo control phase (sequence B-A)	26 (5)	24 (6)	22 (5)
Min-X with open eyes (mm)	Intervention phase (sequence A-B)	-7.75 (2.75)	0.25 (6.00)	-8.25 (4.50)
	Intervention phase (sequence B-A)	-7.00 (7.50)	-7.00 (6.00)	-6.00 (4.50)
	Placebo control phase (sequence A-B)	-6.75 (10.25)	-5.00 (5.50)	-9.00 (9.87)
	Placebo control phase (sequence B-A)	-4.00 (4.50)	0.50 (5.00)	-5.00 (4.50)
Min-Y with open eyes (mm)	Intervention phase (sequence A-B)	-3.75 (5.75)	5.75 (2.37)	-2.75 (8.62)
	Intervention phase (sequence B-A)	-3.00 (5.00)	-3.00 (4.00)	-3.00 (6.00)
	Placebo control phase (sequence A-B)	-2.25 (4.62)	-3.50 (9.87)	-3.75 (6.37)
	Placebo control phase (sequence B-A)	-1.50 (7.50)	-8.50 (7.00)	-5.00 (6.50)
Max-X with open eyes (mm)	Intervention phase (sequence A-B)	-0.25 (5.37)	5.25 (3.00)	-2.50 (5.00)
	Intervention phase (sequence B-A)	-0.50 (11.00)	-2.00 (3.50)	0.00 (5.50)
	Placebo control phase (sequence A-B)	3.00 (18.25)	1.50 (5.75)	2.00 (7.62)
	Placebo control phase (sequence B-A)	1.00 (2.00)	7.00 (5.00)	0.00 (3.50)
Max-Y with open eyes (mm)	Intervention phase (sequence A-B)	7.50 (5.50)	2.25 (4.00)	6.50 (9.62)
	Intervention phase (sequence B-A)	5.50 (9.50)	5.50 (3.50)	5.50 (3.00)
	Placebo control phase (sequence A-B)	7.25 (12.75)	6.00 (4.50)	7.00 (3.87)
	Placebo control phase (sequence B-A)	4.50 (4.50)	-0.50 (6.50)	3.00 (6.00)
Area with open eyes (mm²)	Intervention phase (sequence A-B)	10.00 (8.75)	5.00 (2.37)	7.25 (15.50)
	Intervention phase (sequence B-A)	7.00 (14.50)	3.50 (8.50)	4.50 (4.00)
	Placebo control phase (sequence A-B)	12.75 (60.25)	8.25 (12.25)	10.00 (28.12)
	Placebo control phase (sequence B-A)	5.50 (8.00)	6.50 (6.00)	6.00 (5.00)
Min-X with closed eyes (mm)	Intervention phase (sequence A-B)	-7.75 (4.25)	0.50 (7.00)	-6.50 (4.37)
	Intervention phase (sequence B-A)	-8.00 (6.00)	-7.00 (8.50)	-5.50 (5.50)
	Placebo control phase (sequence A-B)	-8.00 (9.62)	-5.75 (6.12)	-7.75 (6.62)
	Placebo control phase (sequence B-A)	-6.00 (2.00)	2.00 (5.50)	-7.00 (5.50)
Min-Y with closed eyes (mm)	Intervention phase (sequence A-B)	-1.50 (3.87)	5.00 (4.37)	-3.25 (10.12)
	Intervention phase (sequence B-A)	-4.50 (5.50)	-1.00 (4.50)	-3.00 (10.00)
	Placebo control phase (sequence A-B)	-4.75 (4.12)	-4.25 (5.12)	-5.75 (7.75)
	Placebo control phase (sequence B-A)	-7.00 (5.00)	-11.00 (4.50)	-7.50 (9.00)
Max-X with closed eyes (mm)	Intervention phase (sequence A-B)	-1.00 (4.87)	5.75 (7.37)	-1.25 (4.25)

	Intervention phase (sequence B-A)	3.00 (8.00)	-2.00 (6.00)	1.50 (6.00)
	Placebo control phase (sequence A-B)	1.75 (8.12)	0.25 (8.37)	-0.50 (5.62)
	Placebo control phase (sequence B-A)	-1.50 (5.50)	7.00 (5.50)	-1.50 (9.50)
Max-Y with closed eyes (mm)	Intervention phase (sequence A-B)	8.75 (4.50)	2.25 (2.00)	7.25 (4.00)
	Intervention phase (sequence B-A)	7.00 (3.50)	6.00 (5.50)	5.50 (3.50)
	Placebo control phase (sequence A-B)	7.00 (7.37)	5.50 (8.12)	7.00 (5.62)
	Placebo control phase (sequence B-A)	4.00 (6.00)	0.00 (3.50)	7.00 (11.00)
Area with closed eyes (mm^2)	Intervention phase (sequence A-B)	7.50 (8.50)	5.00 (7.75)	6.75 (10.62)
	Intervention phase (sequence B-A)	4.00 (18.50)	5.00 (7.00)	7.00 (16.00)
	Placebo control phase (sequence A-B)	22.00 (29.25)	7.75 (10.50)	14.50 (21.25)
	Placebo control phase (sequence B-A)	7.00 (7.50)	6.50 (11.00)	8.50 (8.50)

Sequence B-A: sequence control placebo- intervention; Sequence A-B: sequence intervention – control placebo; T0: outcome measures at baseline; T1: outcome measures at post-treatment assessment; T2: outcome measures at follow-up assessment.
